# Introduction of Mercaptoethyl at Sorafenib Pyridine-2-Amide Motif as a Potentially Effective Chain to Further get Sorafenib-PEG-DGL

**DOI:** 10.3390/molecules25030573

**Published:** 2020-01-28

**Authors:** Ke Wang, Kudelaidi Kuerbana, Qi Wan, Zhihui Yu, Li Ye, Ying Chen

**Affiliations:** 1 Department of Medicinal Chemistry, School of Pharmacy, Fudan University, Shanghai 201203, China; 16211030008@fudan.edu.cn (K.W.); kudelaidi@fudan.edu.cn (K.K.); 17211030068@fudan.edu.cn (Q.W.); 18211030008@fudan.edu.cn (Z.Y.); 2 Pharmaceutical Sciences Division, School of Pharmacy, University of Wisconsin-Madison, 777 Highland Avenue, Madison, WI 53705-2222,USA; 3 Department of Biological Medicines & Shanghai Engineering Research Center of ImmunoTherapeutics, School of Pharmacy, Fudan University, Shanghai 201203, China

**Keywords:** sorafenib analogs, antiproliferation, nanoparticles (NPs), molecular modeling

## Abstract

The crystal structure of the sorafenib and B-RAF complex indicates that the binding cavity occupied by the pyridine-2-carboxamide in sorafenib has a large variable space, making it a reasonable modification site. In order to identify novel compounds with anti-cancer activity, better safety and polar groups for further application, five sorafenib analogs with new pyridine-2-amide side chains were designed and synthesized. Preliminary pharmacologic studies showed that these compounds displayed much lower toxicities than that of sorafenib. Among them, compound **10b** bearing mercaptoethyl group kept relevant antiproliferation potency compared to sorafenib in Huh7 and Hela cell lines with values of IC_50_ 58.79 and 63.67 μM, respectively. As a small molecule inhibitor targeting protein tyrosine kinases, thiol in compound **10b** would be an active group to react with maleimide in a mild condition for forming nanoparticles Sorafenib-PEG-DGL, which could be developed as a delivery vehicle to improve the concentration of anti-tumor therapeutic agents in the target cancer tissue and reduce side effects in the next study.

Academic Editor: Fawaz Aldabbagh

## 1. Introduction

Protein tyrosine kinases (PTKs) are key enzymes in many signal transduction pathways and play a crucial role in various cell regulatory processes [1,2]. Numerous small molecule inhibitors for PTKs have been developed as antitumor drugs in the past ten years [3]. Sorafenib, approved by the Food and Drug Administration (FDA) in 2005, has shown to be effective against several solid tumors and a standard treatment for HCC (hepatocellular carcinoma) [4] and renal cancer. Sorafenib is a multikinase inhibitor that blocks several targets including Fms-like tyrosine kinase-3 (FLT3), platelet-derived growth factor (PDGF), vascular endothelial growth factor (VEGF), c-Kit and B-RAF signaling [5] in both tumor cells and the surrounding endothelial cells [6,7,8]. The underlying mechanism is believed to involve competitive inhibition of the ATP binding to the catalytic domains of the various kinases [9].

**Figure 1:**
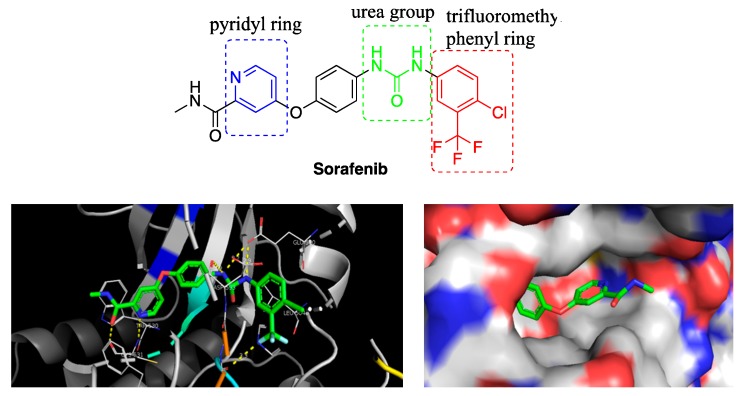
The structure of sorafenib (top) and the binding mode (bottom, left) and binding cavity (bottom, right) of sorafenib and B-RAF kinase.

The crystal structure of sorafenib and B-RAF kinase complex (PDB code: 1UWH) shows that sorafenib spans the length of interfacial cleft of bilobal architecture and buries deep between C and N loop [10]. As shown in Figure 1, the pyridyl ring of sorafenib occupies the ATP-binding pocket, forming a hydrogen bond with Cys531 and interacting with aromatic residues of Trp530 and Phe582. On the other end of sorafenib, the lipophilic trifluoromethyl phenyl ring inserts into a hydrophobic pocket formed between αC and αE helices and *N*-terminal regions of DFG (Asp-Phe-Gly) motif and the catalytic loop. What is more, the urea group forms three hydrogen bonds with catalytic residue Gln500 and the main chain nitrogen of Asp593. The binding mode implies that the pyridyl ring, urea group and trifluoromethyl phenyl ring are key to binding affinity, and the methyl group of amide in the pyridine-2-amide motif may allow to be further modified by other substituents.

**Figure 2:**
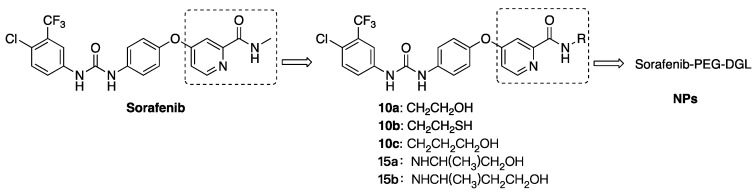
Design of sorafenib derivatives and nanoparticles (NPs).

In 2012, Feng’s group claimed a series of 2-picolinyl hydrazide-containing sorafenib analogs exhibiting better antitumor activity against pancreatic cancer and HCC cell lines than that of sorafenib [11]. The results suggested that the modification of the pyridine-2-amide motif was reasonable, which encouraged us to replace the methyl group of pyridin-2-amide with several alkyl side chains containing hydroxyl or thiol group and synthesize five new derivatives (Figure 2, **10a**–**c** and **15a**–**b**). The research work of this structure optimization aimed at maintaining their strong antiproliferation activity as well as exploring an effective side chain containing OH or SH for preparing NPs (nanoparticles) in the next study. After the structures of **10a**–**c** and **15a**–**b** were confirmed by ^1^H-NMR, ^13^C-NMR and ESI-HRMS spectra, they further were screened for cytotoxicity against seven solid cancer cell lines and two nontumorigenesis cell lines with sorafenib as a control by MTT assays. The results indicated that the length of side chains had moderate influence on the antitumor activity of target compounds. In addition, compound **10b** would be chosen for synthesizing Sorafenib-PEG-DGL in the next study because of its potential antitumor activity and mercaptoethyl group, which could react with maleimide (MAL) in a mild condition.

## 2. Results and Discussion

### 2.1. Chemistry

**Scheme 1:**
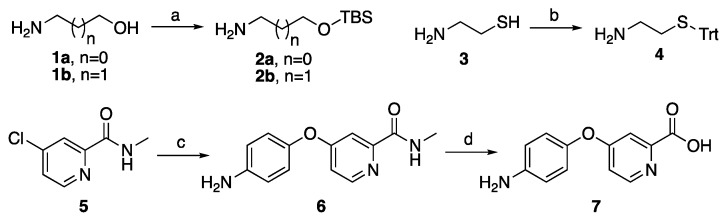
Synthetic routes of **2a**–**b**, **4** and the key intermediate **7**. Reagents and conditions: (**a**) TBSCl, imidazole, dichloromethane (DCM), rt, 1 h, 98%; (**b**) triphenylmethanol, trifluoroacetic acid (TFA), rt, 2 h, 90%; (**c**) 4-amino phenol, (CH_3_)_3_COK, K_2_CO_3_, 80 °C, 3 h, 90% and (**d**) KOH (2.5 mol/L), reflux, 2 h, 90%.

Ethanolamine, 3-aminopropan-1-ol and 2-mercaptoethylamine as side chains were introduced into sorafenib. Meanwhile, as Scheme 1 described, tertbutyldimethylsilane chloride (TBSCl) and triphenylmethanol firstly reacted with OH or SH to get **2a**–**b** and **4** for avoiding the by-products caused by OH or SH group over-reaction. Moreover, the key intermediate **7** was synthesized from starting material *N*-methyl-4-chloropyridine-2-carboxamide (**5**), which reacted with 4-aminophenol to form compound **6** in the presence of potassium tert-butoxide and K_2_CO_3_, following demethylated in 2.5 mol/L KOH in 81% overall yield.

**Scheme 2:**
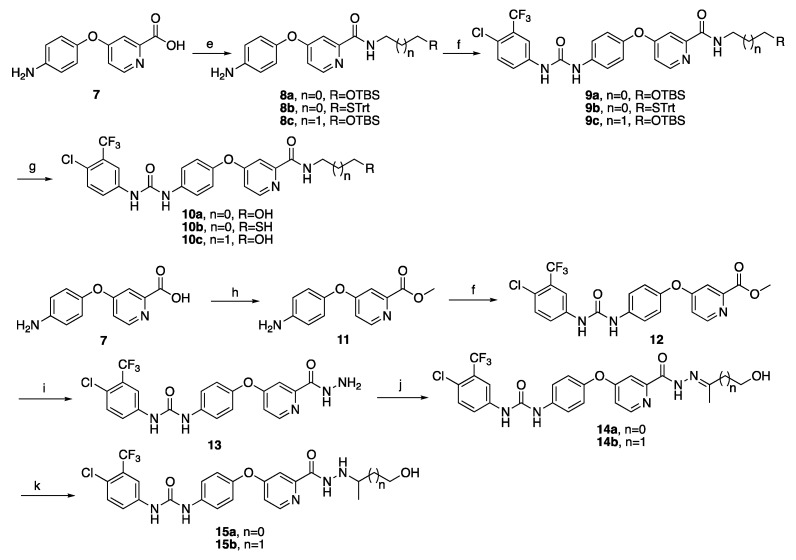
Synthetic routes of target compounds **10a**–**c** and **15a**–**b**. Reagents and conditions: (**e**) **2a**–**b** or **4**, HOBt, EDC^.^HCl, DIEA, DCM, rt, 10 h, 86–88%; (**f**) 4-chloro-3-trifluoromethylphenyl isocyanate, DCM, rt, 2 h, 85–93%; (**g**) I: TBAF, THF, rt, 1 h, 95%; II: TIS, TFA, DCM, rt, 84%. (**h**) MeOH, SOCl_2_, reflux, 8 h, 86%; (**i**) NH_2_NH_2_^.^H_2_O, EtOH, rt, 15 h, 95%; (**j**) Hydroxyacetone or 4-hydroxy-2-butanone, HAc, EtOH, reflux, 8 h, 75% and (**k**) NaBH_3_CN, HAc, MeOH, rt, 8 h, 87%.

The synthesis of target compounds **10a**–**c** and **15a**–**b** starting from the intermediate **7** was depicted in Scheme 2. Compound **7** was treated with **2a**–**b** and **4** to provide intermediates **8a**–**c** in the room temperature, using 1-hydroxybenzotriazole (HOBt), 1-ethyl-3-(3-dimethyllaminopropyl) carbodiimide hydrochloride(EDC^.^HCl) or diisopropyl ethyl amine (DIEA) as condensing agents. Then, **8a**–**c** were reacted with 4-chloro-3-trifluoromethylphenyl isocyanate to give compounds **9a**–**c**, which were deprotected to give target compounds **10a**–**c** under the condition of tetrabutylammonium fluoride (TBAF)/THF or triisopropylsilane (TIS)/TFA. Moreover, the methylation of carboxyl group of **7** with methanol in thionyl chloride afforded ester compound **11**, which was treated with 4-chloro-3-trifluoromethylphenyl isocyanate to form compound **12** in high yield. Then, compound **12** was reacted with hydrazine hydrate to obtain compound **13**, further condensed with hydroxyacetone or 4-hydroxy-2-butanone using acetic acid (HAc) as a catalyst in EtOH to provide intermediates **14a**–**b**. Finally, the reduction of compounds **14a**–**b** with sodium cyanoborohydride (NaBH_3_CN) gave target compounds **15a**–**b**.

### 2.2. In Vitro Antitumor Activities

As shown in Table 1, five target compounds (**10a**–**c** and **15a**–**b)** were tested in seven cancer cells for their effects on cell viability using the MTT assays. These compounds **10a**–**c** bearing amide group at the 2-position of pyridine retained cytotoxic activity against two hepatocellular carcinoma cell lines HepG2 and Huh7. Compound **10b** displayed the best activity against Huh7 with the IC_50_ value of 58.79 μM. What is more, thiol-containing **10b** also exhibited better antitumor activity against MDA-MB-231 (triple negative breast cancer) and Hela (human cervical carcinoma) cell lines than that of **10a** bearing hydroxyl group. Compared to **10a**–**c**, compounds **15a**–**b** containing hydrazine side chain almost lost the antiproliferation activity against Huh7, MCF-7, MDA-MB-231 and Hela cell lines. Especially, compound **15b** having the longest side chain hardly showed the cytotoxic effects on the viability in all tested cancer cell lines. All newly synthesized compounds displayed much weaker activity against mouse melanoma cell line B16F10 compared to sorafenib.

Notably, compounds **10a**–**c** and **15a**–**b** expressed far lower toxicities than sorafenib in HUVEC (Human Umbilical Vein Endothelial Cells) and 293T (Human Adrenal microvascular Endothelial Cells), indicating that these novel compounds have a noteworthy selectivity between tumor and nontumorigenesis cell lines (Table 2).

### 2.3. Molecular Modeling

**Figure 3:**
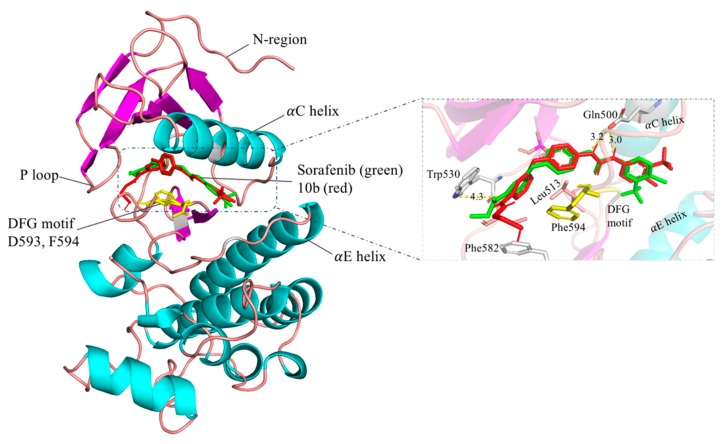
Predicted binding mode of sorafenib and compound **10b** (affinity: −11.2 kcal/mol) in B-RAF kinase. Sorafenib is in green and compound **10b** in red.

Furthermore, we docked compound **10b** into the B-RAF crystal structure (PDB: 1UWH) using Autodock Vina 1.1.2 [12]. The docking mode showed that compound **10b** also inserted into interfacial cleft between the N and C lobes, binding to the same active site as sorafenib (Figure 3). The urea group formed hydrogen bonds with a carboxyl of residue Gln500 and the main chain nitrogen of Asp593. The central phenyl ring of **10b** contacted with the residues Leu513 and Phe594, contributing to the van der Waals interaction. However, the trifluoromethylphenyl ring in compound **10b** distributed closely to the αC helix instead of the hydrophobic pocket that formed between the αC and αE helices and N-terminal regions of the DFG motif, which might explain the slightly decreased activity. Altogether, the binding mode suggested that **10b** could form a strong binding in the active site of B-RAF. Therefore, the compound **10b** bearing thiol group might be as an effective compound to react with maleimide (MAL) in the mild condition for further application [13].

## 3. Materials and Methods

### 3.1. General Information

Chemicals were purchased from the following suppliers: Sinopharm, Adamas and Sigma Aldrich. Solvents were dried before use, if required. Air- and moisture-sensitive reactions were carried out under nitrogen atmosphere. Room temperature (rt) refers to 20–25 °C. The progress of a reaction was monitored by thin layer chromatography (TLC) using pre-coated TLC sheets purchased from Sinopharm. Detected spots were observed under UV light at λ 254 nm and 365 nm. Melting points were measured on a SGW X-4 microscopy melting point apparatus without correction. ^1^H spectral data were recorded with a Varian Mercury Plus 400 MHz spectrometer and ^13^C-NMR spectral data were recorded with a Bruker DRX 600MHz spectrometer, both at 303 K using TMS as an internal standard. All chemical shifts were reported in ppm (*δ*) and coupling constants (*J*) were in hertz (Hz). Mass spectra were recorded on Agilent Technologies 1260 infinity LC/MS instrument. The chromatograms were conducted on silica gel (100−200 mesh) and visualized under UV light at λ 254 and 365 nm (Supplementary Materials).

### 3.2. Synthesis

General Procedure for the Preparation of **2a**–**b.** To dimethyl isopropyl chlorosilane (TBSCl, 3.15 g, 21.0 mmol) in dichloromethane (DCM, 10 mL) was added to a mixture of ethanolamine or 3-aminopropan-1-ol (20.0 mmol) and imidazole (2.72 g, 40.0 mmol) in DCM (40 mL) in room temperature. The mixture was reacted for 3 h and then poured into water (60 mL), extracted with DCM (3 × 30 mL). The combined organic layers were washed with brine, dried over Na_2_SO_4_, filtered and the filtrate was concentrated. The residue was dried to give **2a**–**b**.

*2-((Tert-butyldimethylsilyl)oxy)ethan-1-amine* (**2a**): The title compound was obtained starting from ethanolamine (**1a**). Analytical data for **2a** (colorless liquid, 98% yield): ^1^H-NMR (400 MHz, CDCl_3_) *δ* 3.68–3.58 (m, 2H, NH_2_CH_2_CH_2_O-), 2.78 (dd, 2H, NH_2_CH_2_CH_2_O-, *J* = 9.4, 4.6 Hz), 0.91 (s, 9H, -SiC(CH_3_)_3_), 0.07 (s, 6H, -Si(CH_3_)_2_).

*3-((Tert-butyldimethylsilyl)oxy)propan-1-amine* (**2b**): The title compound was obtained starting from 3-aminopropan-1-ol (**1b**). Analytical data for **2b** (colorless liquid, 99% yield): ^1^H-NMR (400 MHz, CDCl_3_) *δ* 3.70 (t, 2H, -CH_2_CH_2_O-, *J* = 6.0 Hz), 2.80 (t, 2H, NH_2_CH_2_-, *J* = 6.7 Hz), 1.71-1.61 (m, 2H, -CH_2_CH_2_CH_2_O-), 0.89 (s, 9H, -SiC(CH_3_)_3_), 0.05 (s, 6H, -Si(CH_3_)_2_).

*2-(Tritylthio)ethanamine* (**4**): Triphenylmethyl (**3**, Trt, 2.29 g, 8.8 mmol) was added to a solution of 2-mercaptoethylamine (1.0 g, 8.8 mmol) in trifluoroacetate (TFA, 10 mL) in room temperature. The mixture was reacted for 1 h and turned to red. Then, it was poured into water (40 mL), extracted with ethyl acetate (EA, 3 × 20 mL). The combined organic layers were washed with brine, dried over Na_2_SO_4_, filtered and the filtrate was concentrated. A white precipitate was filtered and recrystallized from EA to produce **4** (white solid, 90% yield, mp 93–95 °C). ESI-MS *m*/*z* 320.2 [M + H]^+^; ^1^H-NMR (400 MHz, CDCl_3_) *δ* 7.74 (brs, 2H, -NH_2_), 7.41 (d, 5H, ArH, *J* = 7.3 Hz), 7.27 (m, 6H, ArH), 7.22–7.17 (m, 4H, ArH), 2.59 (t, 2H, -CH_2_CH_2_S-, *J* = 6.6 Hz), 2.26-2.19 (m, 2H, NH_2_CH_2_CH_2_-).

*N-methyl-4-(4-aminophenoxy)picolinamide* (**6**): Potassium tert-butoxide (1.4 g, 11.3 mmol) was added to a solution of 4-aminophenol (1.0 g, 9.1 mmol) in DMF (15 mL) under nitrogen atmosphere. The mixture was stirred for 3 h in room temperature and *N*-methyl-4-chloropyridine-2-carboxamide (**5**, 1.2 g, 7.1 mmol) and K_2_CO_3_ (0.6 g, 4.2 mmol) was added. Then, the suspension was stirred at 80 °C for 3 h and poured into water (40 mL), extracted with ethyl acetate (EA, 3 × 30 mL). The combined organic layers were washed with brine, dried over Na_2_SO_4_, filtered and the filtrate was concentrated to afford **6** (yellow oil, 92% yield). ESI-MS *m*/*z* 244.2 [M + H]^+^; ^1^H-NMR (400 MHz, CDCl_3_) *δ* 8.33 (d, 1H, ArH, *J* = 5.5 Hz,), 8.01 (brs, 1H, -CONHCH_3_), 7.67 (d, 1H, ArH, *J* = 1.9 Hz), 6.92 (dd, 1H, ArH, *J*_1_ = 5.5 Hz, *J*_2_ = 2.4 Hz), 6.88 (d, 2H, ArH, *J* = 8.6 Hz), 6.71 (d, 2H, ArH, *J* = 8.6 Hz), 3.00 (d, 3H, -NHCH_3_, *J* = 5.1 Hz).

*4-(4-Aminophenoxy)-2-pyridine carboxylic acid* (**7**): To a stirred solution of K_2_CO_3_ (2.5 mol/L, 20 mL), **6** (1.70 g, 7.0 mmol) was added. After 5 h of refluxing reaction, the solution was carefully adjusted to pH 5 by the addition of 2N HCl. The reaction mixture was evaporated and the residue was purified by column chromatography on silica gel (100−200 mesh, and visualized under UV light at λ 254 and 365 nm; eluent, DCM/MeOH 5:1) to give **7** (white solid, 90% yield, mp 207–209 °C). ESI-MS *m*/*z* 231.1 [M + H]^+^; ^1^H-NMR (400 MHz, DMSO-*d*_6_) *δ* 8.52 (d, 1H, ArH, *J* = 4.2 Hz), 7.36 (s, 1H, ArH), 7.12 (s, 1H, ArH), 6.88 (d, 2H, ArH, *J* = 7.6 Hz), 6.64 (d, 2H, ArH, *J* = 7.5 Hz).

General Procedure for the Preparation of **8a**–**c**. 1-Hydroxybenzotriazole (HOBt, 100 mg, 0.7 mmol), 3-(3-dimethylaminopropyl)-1-ethylcarbodiimide hydrochloride (EDC^.^HCl, 150 mg, 0.78 mmol) and diisopropylamine (0.4 mL, 2.34 mmol) were combined in a stirred mixture of **7** (100 mg, 0.43 mmol) in DCM (15 mL). After adding compound **2a**–**b** or **4** and stirring reaction for 10 h at room temperature, the mixture was poured into water (30 mL), extracted with DCM (3 × 15 mL). The combined organic layers were washed with brine, dried over Na_2_SO_4_, filtered and the filtrate was concentrated. The residue was purified by column chromatography on silica gel (100−200 mesh, and visualized under UV light at λ 254 and 365 nm; eluent, PE/EA) to give **8a**–**c**.

*N-(2-((tert-butyldimethylsilyl)oxy)ethyl)-4-(4-aminophenoxy)picolinamide* (**8a**): The title compound was obtained starting from **2a** and **7**. Analytical data for **8a** (yellow liquid, 86% yield): ESI-MS *m*/*z* 388.2 [M + H]^+^; ^1^H-NMR (400 MHz, CDCl_3_) *δ* 8.35 (d, 2H, ArH, *J* = 5.6 Hz), 7.66 (brs, 1H, -CONH-), 6.89 (d, 2H, ArH, *J* = 8.8 Hz), 6.71 (d, 2H, ArH, *J* = 8.7 Hz), 3.77 (t, 2H, -CH_2_CH_2_O-, *J* = 5.3 Hz), 3.70 (s, 2H, -NH_2_), 3.56 (dd, 2H, -NHCH_2_CH_2_-, *J*_1_ = 10.3 Hz, *J*_2_ = 4.6 Hz), 0.91 (s, 9H, -SiC(CH_3_)_3_), 0.07 (s, 6H, -Si(CH_3_)_2_).

*N-(2-(tritylthio)ethyl)-4-(4-aminophenoxy)picolinamide* (**8b**): The title compound was obtained starting from **4** and **7**. Analytical data for **8b** (yellow liquid, 88% yield): ESI-MS *m*/*z* 554.2 [M + H]^+^; ^1^H-NMR (400 MHz, CDCl_3_) *δ* 8.34 (d, 1H, ArH, *J* = 5.6 Hz), 8.13 (t, 1H, -CONH-, *J* = 5.7 Hz), 7.61 (d, 1H, ArH, *J* = 1.3 Hz), 7.42 (d, 5H, ArH, *J* = 7.6 Hz), 7.28–7.17 (m, 10H, ArH), 6.93 (dd, 1H, ArH, *J*_1_ = 5.6 Hz, *J*_2_ = 2.5 Hz), 6.88 (d, 2H, ArH, *J* = 9.4 Hz), 6.70 (d, 2H, ArH, *J* = 8.7 Hz), 3.70 (brs, 2H, -NH_2_), 3.28 (q, 2H, -NHCH_2_-, *J* = 6.6 Hz), 2.48 (t, 2H, -CH_2_CH_2_S-, *J* = 6.6 Hz).

*N-(3-((tert-butyldimethylsilyl)oxy)propyl)-4-(4-aminophenoxy)picolinamide* (**8c**): The title compound was obtained starting from **2b** and **7**. Analytical data for **8c** (yellow liquid, 84% yield): ESI-MS *m*/*z* 402.2 [M + H]^+^; ^1^H-NMR (400 MHz, CDCl_3_) *δ* 8.32 (d, 2H, ArH, *J* = 5.6 Hz), 7.65 (s, 1H, ArH), 6.88 (d, 2H, ArH, *J* = 8.9 Hz), 6.71 (d, 2H, ArH, *J* = 8.3 Hz), 4.68 (brs, 2H, -NH_2_), 3.77 (t, 2H, -CH_2_CH_2_O-, *J* = 5.7 Hz), 3.55 (q, 2H, -NHCH_2_CH_2_-, *J* = 6.0 Hz), 1.88–1.77 (m, 2H, -CH_2_CH_2_CH_2_-), 0.91 (s, 9H, -SiC(CH_3_)_3_), 0.08 (s, 6H, -Si(CH_3_)_2_).

General Procedure for the Preparation of **9a**–**c** and **12**. A mixture of compound **8a**–**c** or **11** (1.5 mmol) in DCM (10 mL) was stirred at room temperature, while a solution of 4-chloro-3-trifluoromethylphenyl isocyanate (1.5 mmol) in DCM (5 mL) was slowly added in ice-bath condition. Afterwards, stirring was continued for 2 h at room temperature. The reaction mixture was poured into water (30 mL), extracted with DCM (3 × 15 mL). The combined organic layers were washed with brine, dried over Na_2_SO_4_, filtered and the filtrate was concentrated. The residue was purified by column chromatography on silica gel (eluent, PE/EA) to give **9a**–**c** and **12**.

*N-(2-((tert-butyldimethylsilyl)oxy)ethyl)-4-(4-(3-(4-chloro-3-(trifluoromethyl)phenyl)ureido)phenoxy) picolinamide* (**9a**): The title compound was obtained starting from **8a**. Analytical data for **9a** (white solid, 86% yield, mp 133–135 °C): ESI-MS *m*/*z* 610.1 [M + H]^+^; ^1^H-NMR (400 MHz, CDCl_3_) *δ* 8.75 (s, 1H, -CONH-), 8.48 (d, 1H, ArH, *J* = 5.7 Hz), 8.45 (s, 1H, -NHCONH-), 8.19 (s, 1H, -NHCONH-), 7.66 (d, 2H, ArH, *J* = 8.5 Hz), 7.52 (s, 1H, ArH), 7.34 (dd, 2H, ArH, *J*_1_ = 16.6 Hz, *J*_2_ =8.1 Hz), 7.27 (t, 1H, ArH, *J* = 3.4 Hz), 7.18 (s, 1H, ArH), 6.97 (d, 2H, ArH, *J* = 7.7 Hz), 3.79 (t, 2H, -CH_2_CH_2_O-, *J* = 4.4 Hz), 3.57 (dd, 2H, -NHCH_2_-, *J*_1_ = 9.5 Hz, *J*_2_ = 4.5 Hz), 0.90 (s, 9H, -SiC(CH_3_)_3_), 0.06 (s, 6H, -Si(CH_3_)_2_).

*N-(*2-(tritylthio)ethyl*)-4-(4-(3-(4-chloro-3-(trifluoromethyl)phenyl)ureido)phenoxy)picolinamide* (**9b**): The title compound was obtained starting from **8b**. Analytical data for **9b** (white solid, 93% yield, mp 107–109 °C): ESI-MS *m*/*z* 754.2 [M + H]^+^; ^1^H-NMR (400 MHz, CDCl_3_) *δ* 8.48 (d, 2H, ArH, *J* = 1.6 Hz), 8.08 (s, 1H, -NHCONH-), 7.93 (s, 1H, -NHCONH-), 7.69 (s, 1H, -CONH-), 7.48 (dd, 3H, ArH, *J*_1_ = 12.3 Hz, *J*_2_ = 8.6 Hz), 7.35 (m, 5H, ArH), 7.29 (d, 2H, ArH, *J* = 2.6 Hz), 7.23–7.15 (m, 10H, ArH), 6.95 (d, 2H, ArH, *J* = 4.0 Hz), 3.28–3.23 (m, 2H, -NHCH_2_-), 2.48–2.43 (m, 2H, -CH_2_CH_2_S-).

*N-(3-((tert-butyldimethylsilyl)oxy)propyl)-4-(4-(3-(4-chloro-3-(trifluoromethyl)phenyl)ureido)phenoxy) picolinamide* (**9c**): The title compound was obtained starting from **8c**. Analytical data for **9c** (white solid, 90% yield, mp 118–120 °C): ESI-MS *m*/*z* 624.2 [M + H]^+^; ^1^H-NMR (400 MHz, CDCl_3_) *δ* 8.83 (d, 1H, -CONH-, *J* = 5.5 Hz), 8.53 (s, 1H, -NHCONH-), 8.44 (d, 1H, ArH, *J* = 5.3 Hz), 8.24 (s, 1H, -NHCONH-), 7.70 (d, 2H, ArH, *J* = 7.4 Hz), 7.54 (s, 1H, ArH), 7.41–7.34 (m, 3H, ArH), 7.15 (s, 1H, ArH), 6.99 (t, 2H, ArH, *J* = 7.0 Hz), 3.78 (dd, 2H, -CH_2_CH_2_O-, *J*_1_ = 12.1 Hz, *J*_2_ = 5.4 Hz), 3.58 (dd, 2H, -NHCH_2_-, *J*_1_ = 12.8 Hz, *J*_2_ = 7.6 Hz), 1.87–1.81 (m, 2H, -CH_2_CH_2_CH_2_O-), 0.90 (s, 9H, -SiC(CH_3_)_3_), 0.08 (s, 6H, -Si(CH_3_)_2_).

*Methyl 4-(4-(3-(4-chloro-3-(trifluoromethyl)phenyl)ureido)phenoxy)picolinate* (**12**): The title compound was obtained starting from **11**. Analytical data for **12** (white solid, 76% yield, mp 172–174 °C): ESI-MS *m*/*z* 466.2 [M + H]^+^; ^1^H-NMR (400 MHz, CDCl_3_) *δ* 8.52–8.44 (m, 2H, ArH), 8.40 (s, 1H, ArH), 7.69 (s, 2H, -NHCONH-), 7.63 (d, 1H, ArH, *J* = 8.5 Hz), 7.45 (d, 2H, ArH, *J* = 8.6 Hz), 7.36 (d, 1H, ArH, *J* = 8.6 Hz), 7.00 (d, 2H, ArH, *J* = 8.7 Hz), 6.97 (dd, 1H, ArH, *J*_1_ = 4.9 Hz, *J*_2_ = 2.4 Hz), 4.02 (s, 3H, -COOCH_3_).

General Procedure for the Preparation of **10a** and **10c**. Tetrabutylammonium fluoride trihydrate (416 mg, 1.3 mmol) was added to a solution of **9a** or **9c** (1.3 mmol) in tetrahydrofuran (10 mL). The mixture was stirred at room temperature for 1 h and then poured into water (30 mL), extracted with DCM (3 × 15 mL). The combined organic layers were washed with brine, dried over Na_2_SO_4_, filtered and the filtrate was concentrated. The residue was purified by column chromatography on silica gel (100−200 mesh, and visualized under UV light at λ 254 and 365 nm; eluent, PE/EA) to offer **10a** and **10c**.

*N-(2-hydroxyethyl)-4-(4-(3-(4-chloro-3-(trifluoromethyl)phenyl)ureido)phenoxy)picolinamide* (**10a**): The title compound was obtained starting from **9a**. Analytical data for **10a** (white solid, 96% yield, mp 193–195 °C): ESI-MS *m*/*z* 495.1 [M + H]^+^; ^1^H-NMR (400 MHz, DMSO-*d*_6_) *δ* 9.24 (s, 1H, -NHCONH-), 9.03 (s, 1H, -NHCONH-), 8.71 (t, 1H, -CONH-, *J* = 5.9 Hz), 8.52 (d, 1H, ArH, *J* = 5.6 Hz), 8.13 (s, 1H, ArH), 7.70-7.57 (m, 4H, ArH), 7.38 (d, 1H, ArH, *J* = 2.0 Hz), 7.18 (d, 3H, ArH, *J* = 8.6 Hz), 4.80 (t, 1H, -OH, *J* = 5.4 Hz), 3.50 (q, 2H, -CH_2_CH_2_OH, *J* = 5.8 Hz), 3.38–3.31 (m, 2H, -CH_2_CH_2_OH). ^13^C-NMR (151 MHz, DMSO-*d*_6_) *δ* 165.93, 163.10, 152.37, 152.17, 150.27, 147.75, 139.23, 136.96, 131.90, 126.72, 126.51, 123.62, 123.02, 122.24, 121.34, 120.42, 116.73, 114.09, 108.61, 59.48, 41.50. ESI-HRMS (*m*/*z*) [M + H]^+^ calcd for C_22_H_18_ClF_3_N_4_O_4_ 459.1041, obsd 459.1046, ppm error 0.9.

*N-(3-hydroxypropyl)-4-(4-(3-(4-chloro-3-(trifluoromethyl)phenyl)ureido)phenoxy)picolinamide* (**10c**): The title compound was obtained starting from **9c**. Analytical data for **10c** (white solid, 96% yield, mp 103–105 °C): ESI-MS *m*/*z* 509.2 [M + H]^+^; ^1^H-NMR (400 MHz, DMSO-*d*_6_) *δ* 9.98 (s, 1H, -NHCONH-), 9.64 (s, 1H, -NHCONH-), 8.83 (t, 1H, -CONH-, *J* = 4.4 Hz), 8.50 (d, 1H, ArH, *J* = 5.4 Hz), 8.12 (s, 1H, ArH), 7.64 (d, 2H, ArH, *J* = 7.7 Hz), 7.59 (d, 2H, ArH, *J* = 8.2 Hz), 7.39 (s, 1H, ArH), 7.20–7.11 (m, 3H, ArH), 4.55 (t, 1H*,* -OH, *J* = 4.7 Hz), 3.44 (dd, 2H, -CH_2_CH_2_OH, *J*_1_ = 11.0 Hz, *J*_2_ = 5.6 Hz), 3.32 (t, 2H, -NHCH_2_-, *J* = 4.5 Hz), 1.70-1.60 (m, 2H, -CH_2_CH_2_OH). ^13^C-NMR (151 MHz, DMSO-*d*_6_) *δ* 165.92, 163.08, 152.45, 152.35, 150.27, 147.70, 139.32, 137.04, 131.93, 126.74, 126.54, 123.64, 122.84, 122.14, 121.83, 121.36, 120.23, 116.54, 113.96, 108.68, 58.68, 36.44, 32.03. ESI-HRMS (*m*/*z*) [M + H]^+^ calcd for C_23_H_20_ClF_3_N_4_O_4_ 509.1198, obsd 509.1203, ppm error 1.1.

*N-(*2-mercaptoethyl*)-4-(4-(3-(4-chloro-3-(trifluoromethyl)phenyl)ureido)phenoxy) picolinamide* (**10b**): Compound **9b** (100 mg, 0.13 mmol) was dissolved in DCM (15 mL). Triisopropylsilane (95 mg, 0.6 mmol) and TFA (0.3 mmol) were added and the mixture was stirred at room temperature overnight. The mixture was extracted with DCM (3 × 20 mL). The combined organic layers were washed with brine, dried over Na_2_SO_4_, filtered and the filtrate was concentrated. The residue was purified by column chromatography on silica gel (eluent, DCM/MeOH 10:1) to offer **10b** (white solid, 84% yield, mp 129–131 °C). ESI-MS *m*/*z* 511.1 [M + H]^+^; ^1^H NMR (400 MHz, DMSO-*d*_6_) *δ* 10.41 (s, 1H, -NHCONH-), 9.99 (s, 1H, -NHCONH-), 9.02-8.99 (m, 1H, -CONH-), 8.52 (dd, 1H, ArH, *J* = 5.4, 2.4 Hz), 8.12 (d, 2H, ArH, *J* = 1.2 Hz), 7.65 (s, 2H, ArH), 7.59 (dd, 2H, ArH, *J* = 8.7, 2.4 Hz), 7.41 (t, 1H, ArH, *J* = 2.7 Hz), 7.20-7.13 (m, 3H, ArH), 4.66 (t, 1H, -SH, *J* = 4.7 Hz), 3.49 (d, 2H, -CH_2_SH, *J* = 4.6 Hz), 2.63 (dd, 2H, -NHCH_2_-, *J*_1_ = 14.6 Hz, *J*_2_ = 5.7 Hz). ^13^C-NMR (151 MHz, DMSO-*d*_6_) *δ* 165.94, 163.21, 158.03, 157.83, 152.57, 152.10, 150.31, 147.54, 139.51, 137.24, 131.95, 122.54, 121.92, 121.36, 119.89, 118.23, 116.24, 114.02, 108.90, 72.16, 60.13. ESI-HRMS (*m*/*z*) [M + H]^+^ calcd for C_22_H_18_ClF_3_N_4_O_3_S 511.0813, obsd 511.0824, ppm error 2.2.

*Methyl 4-(4-aminophenoxy)picolinate* (**11**): To compound **7** (1.0 g, 4.3 mmol) in methanol (30 mL) was added dichlorosulfoxide (1.1 mL) slowly in an ice-bath. The reaction mixture was stirred and refluxed overnight. The reaction mixture was concentrated under reduced pressure then extracted with ethyl acetate (3 × 30 mL). The organic layer was washed with brine, dried over anhydrous Na_2_SO_4_, filtered and concentrated under reduced pressure to afford **11** (white solid, 84% yield, mp 113–115 °C). ESI-MS *m*/*z* 245.1 [M + H]^+^; ^1^H-NMR (400 MHz, CDCl_3_) *δ* 8.53 (d, 1H, ArH, *J* = 5.7 Hz), 7.62 (d, 1H, ArH, *J* = 2.2 Hz), 6.97 (dd, 1H, ArH, *J*_1_ = 5.6 Hz, *J*_2_ = 1.0 Hz), 6.89 (d, 2H, ArH, *J* = 8.7 Hz), 6.73 (d, 2H, ArH, *J* = 8.7 Hz), 3.97 (s, 3H, -COOCH_3_).

*1-(4-Chloro-3-(trifluoromethyl)phenyl)-3-(4-((2-(hydrazinecarbonyl)pyridin-4-yl)oxy)phenyl)urea* (**13**): A mixture of **12** (500 mg, 1.1 mmol) and hydrazine hydrate (0.16 mL) in ethanol (10 mL) was stirred at 90 °C for 2 h. The reaction mixture was concentrated under reduced pressure then extracted with ethyl acetate (3 × 30 mL). The organic layer was washed with brine, dried over anhydrous Na_2_SO_4_, filtered and concentrated under reduced pressure. The solid residue was recrystallized from ethanol to afford **13** (gray solid, 95% yield, mp 119-121 °C). ESI-MS *m*/*z* 466.1 [M + H]^+^; ^1^H-NMR (400 MHz, DMSO-*d*_6_) *δ* 9.93 (s, 1H, -CONHNH_2_), 9.25 (s, 1H, -NHCONH-), 9.04 (s, 1H, -NHCONH-), 8.49 (d, 1H, ArH, *J* = 5.5 Hz), 8.13 (s, 1H, ArH), 7.68–7.58 (m, 4H, ArH), 7.35 (t, 1H, ArH, *J* = 5.0 Hz), 7.19 (d, 2H, ArH, *J* = 8.8 Hz), 7.13 (dd, 1H, ArH, *J*_1_ = 5.3Hz, *J*_2_ = 2.4 Hz), 4.56 (brs, 2H, -CONHNH_2_).

General Procedure for the Preparation of **14a**–**b.** A mixture of **13** (50 mg, 0.11 mmol) and hydroxyacetone (10 μL, 0.15 mmol) or 4-hydroxy-2-butanone (10 μL, 0.15 mmol) in ethanol (8 mL) was stirred at 90 °C for 24h, using acetic acid as a catalyst. The reaction mixture was concentrated under reduced pressure. The residue was purified by recrystallization from ethanol or methanol to afford **14a**–**b**.

(E)-1-(4-Chloro-3-(trifluoromethyl)phenyl)-3-(4-((2-(2-(1-hydroxypropan-2-ylidene)hydrazine-1-carbonyl)pyridin-4-yl)oxy)phenyl)urea (**14a**): The title compound was obtained starting from hydroxyacetone. Analytical data for **14a** (white solid, 75% yield, mp 198–200 °C): ESI-MS *m*/*z* 522.2 [M + H]^+^; ^1^H NMR (400 MHz, DMSO-d_6_, ratio 73:27, the minor signals are marked with an asterisk, mixture signals are marked with double asterisks) δ 12.68 (s, 0.73H), 10.77* (s, 0.22H), 9.25** (s, 1H), 9.03** (s, 1H), 8.57* (d, 0.29H, J = 5.5 Hz), 8.51 (d, 0.62H, J = 5.6 Hz), 8.13** (s, 1H), 7.73–7.55** (m, 4H), 7.42** (s, 1H), 7.20** (d, 3H, J = 7.0 Hz), 6.11 (t, 0.68H, J = 4.9 Hz), 5.24* (t, 0.23H, J = 5.9 Hz), 4.34 (d, 1.49H, J = 4.8 Hz), 4.04* (d, 0.54H, J = 6.1 Hz), 1.98* (s, 0.86H), 1.91 (s, 2.14H).

(E)-1-(4-Chloro-3-(trifluoromethyl)phenyl)-3-(4-((2-(2-(4-hydroxybutan-2-ylidene)hydrazine-1-carbonyl)pyridin-4-yl)oxy)phenyl)urea (**14b**): The title compound was obtained starting from 4-hydroxy-2-butanone. Analytical data for **14b** (white solid, 70% yield, mp 205–207 °C): ESI-MS *m*/*z* 536.2 [M + H]^+^; ^1^H-NMR (400 MHz, DMSO-d_6_, ratio 75:25, the minor signals are marked with an asterisk, mixture signals are marked with double asterisks) δ 11.47 (s, 0.75H), 10.73* (s, 0.25H), 9.25** (s, 1H), 9.03** (s, 1H), 8.54** (dd, 1H, J = 13.9, 5.8 Hz), 8.13** (s, 1H), 7.64** (dt, 4H, J = 17.9, 8.8 Hz), 7.41** (d, 1H, J = 2.5 Hz), 7.20** (d, 3H, J = 8.6 Hz), 5.36 (t, 0.75H, J = 4.4 Hz), 4.62* (t, 0.25H, J = 5.4 Hz), 3.76–3.60** (m, 2H), 2.50–2.45** (m, 2H), 2.04 (s, 2.33H), 1.98* (s, 0.54H). ^13^C-NMR (151 MHz, DMSO-d_6_) δ 166.00, 160.49, 159.09*, 152.38, 151.96*, 150.49, 147.68, 139.24, 137.02, 131.91, 126.72, 126.52*, 123.63, 123.03*, 122.26, 121.82, 121.38*, 120.46, 116.74, 114.36, 108.85, 58.28*, 57.67, 41.71*, 34.30, 23.23, 15.67*. ESI-HRMS (*m*/*z*) [M + H]^+^ calcd for C_24_H_21_ClF_3_N_5_O_4_ 536.1307, obsd 536.1312, ppm error 1.0.

General Procedure for the Preparation of **15a**–**b.** To a stirred solution of **14a**–**b** (0.057 mmol) in Methanol (5 mL) at room temperature was added sodium cyanoborohydride (7.6 mg, 0.11 mmol) and acetic acid (3.3 μL). The mixture was reacted at room temperature overnight and then poured into water (10 mL), extracted with ethyl acetate (3 × 15 mL). The combined organic layers were washed with brine, dried over Na_2_SO_4_, filtered and the filtrate was concentrated. The residue was purified by column chromatography on silica gel (100−200 mesh, and visualized under UV light at λ 254 and 365 nm; eluent, DCM/MeOH) to give **15a**–**b**.

*1-(4-Chloro-3-(trifluoromethyl)phenyl)-3-(4-((2-(2-(1-hydroxypropan-2-yl)hydrazine-1-carbonyl)pyridin-4-yl)oxy)phenyl)urea* (**15a**): The title compound was obtained starting from **14a**. Analytical data for **15a** (white solid, 87% yield, mp 139–141 °C): ESI-MS *m*/*z* 524.2 [M + H]^+^; ^1^H-NMR (400 MHz, DMSO-*d*_6_) *δ* 10.14 (brs, 1H, -CONH-), 9.49 (brs, 1H, -NHCONH-), 9.25 (brs, 1H, -NHCONH-), 8.50 (dd, 1H, ArH, *J*_1_ = 5.4 Hz, *J*_2_ = 1.6 Hz), 8.13 (s, 1H, ArH), 7.69-7.56 (m, 4H, ArH), 7.35 (s, 1H, ArH), 7.18 (dd, 3H, ArH, *J*_1_ = 9.1 Hz, *J*_2_ = 1.4 Hz), 5.13 (brs, 1H, -OH), 4.65 (t, 1H, -NHCH-, *J* = 5.3 Hz), 3.33–3.25 (m, 2H, -CHCH_2_OH), 2.96 (d, 1H, -NHCH-, *J* = 4.3 Hz), 0.95 (d, 3H, =CHCH_3_, *J* = 5.0 Hz). ^13^C-NMR (151 MHz, DMSO-*d*_6_) *δ* 165.83, 161.76, 152.46, 151.80, 150.45, 147.66, 139.35, 137.08, 131.91, 126.72, 123.63, 122.90, 122.13, 121.83, 121.35, 120.28, 116.60, 113.98, 108.83, 63.84, 56.44, 15.38. ESI-HRMS (*m*/*z*) [M + H]^+^ calcd for C_23_H_21_ClF_3_N_5_O_4_ 524.1307, obsd 524.1311, ppm error 0.8.

*1-(4-Chloro-3-(trifluoromethyl)phenyl)-3-(4-((2-(2-(4-hydroxybutan-2-yl)hydrazine-1-carbonyl)pyridin-4-yl)oxy)phenyl)urea* (**15b**): The title compound was obtained starting from **14b**. Analytical data for **15b** (white solid, 87% yield, mp 89–92 °C): ESI-MS *m*/*z* 538.2 [M + H]^+^; ^1^H-NMR (400 MHz, DMSO-*d*_6_) *δ* 10.06 (d, 1H, -CONH-, *J* = 7.0 Hz), 9.24 (s, 1H, -NHCONH-), 9.02 (s, 1H, -NHCONH-), 8.51 (d, 1H, ArH, *J* = 5.6 Hz), 8.13 (s, 1H, ArH), 7.64 (d, 2H, ArH, *J* = 6.1 Hz), 7.60 (d, 2H, ArH, *J* = 8.7 Hz), 7.35 (s, 1H, ArH), 7.19 (d, 2H, ArH, *J* = 8.8 Hz), 7.16 (dd, 1H, ArH, *J*_1_ = 5.5 Hz, *J*_2_ = 2.1 Hz), 4.97 (t, 1H, -OH, *J* = 6.0 Hz), 4.44 (t, 1H, -CH_2_CH_2_O-, *J* = 5.0 Hz), 3.58–3.38 (m, 1H, -CH_2_CH_2_O-), 3.09–2.95 (m, 1H, -NHCHCH_3_), 1.63 (m, 1H, -CHCH_2_CH_2_-), 1.43–1.31 (m, 1H, -CHCH_2_CH_2_-), 0.98 (d, 3H, =CHCH_3_, *J* = 6.3 Hz). ^13^C-NMR (151 MHz, DMSO-*d*_6_) *δ* 165.82, 161.81, 152.38, 151.92, 150.44, 147.73, 139.24, 136.98, 131.91, 126.73, 126.52, 123.63, 123.03, 122.25, 121.82, 121.37, 120.43, 116.76, 113.99, 108.77, 58.26, 52.86, 37.53, 18.70. ESI-HRMS (*m*/*z*) [M + H]^+^ calcd for C_24_H_23_ClF_3_N_5_O_4_ 538.1463, obsd 538.1464, ppm error 0.1.

### 3.3. In Vitro Anti-Proliferative Assay

The in vitro anti-proliferation of the chemical compounds was measured by the MTT reagent. Briefly, 5 × 10^3^ cells in 100 μL of medium per well were plated in 96-well plates. After being incubated for 24 h at 37 °C, the cells were treated with different concentration of tested compound or DMSO (as negative control) for 48 h. Then the medium with compound or DMSO was replaced with 200 μL of fresh medium containing 10% MTT (5 mg/mL in PBS) in each well and incubated at 37 °C for 4 h. Last, the MTT-containing medium was discarded and 150 μL of DMSO per well was added to dissolve the formazan crystals newly formed. Absorbance of each well was determined by a microplate reader (Synergy H4, Bio-Tek) at a 570 nm wavelength. The inhibition rates of proliferation were calculated with the following equation:

Inhibition ratio (%) = (OD_DMSO_−OD_compd_)/(OD_DMSO_−OD_blank_) × 100 (1)

The concentration of the compounds that inhibited cell growth by 50% (IC_50_) was calculated using GraphPad Prism, version 6.0.

## 4. Conclusions

Based on sorafenib and B-ARF crystal complex, there is a space for the modification of the 2-pyridine motif of sorafenib using alkyl chains to develop new anti-cancer agents. In order to introduce active side chains including thiol or hydroxyl for the synthesis of Sorafenib-PEG-DGL in the next study, five novel sorafenib derivatives (**10a**–**b**, **15a**–**b**) with oxygen or sulphur alkyl side-chain on pyridine-2-amide scaffold were prepared and evaluated their anti-cancer activities in vitro. The preliminary structure and activity relationships indicated that pyridine-2-amide was essential for antitumor activity and pyridine-2-hydrazine led to the loss of activity. The length of side chain had moderate influence on the antiproliferation activity against tumor cells. Among them, compound **10b** bearing thiol alkyl group showed a good antiproliferation activity, which provided us a promising candidate for the future preparing nanoparticles. Moreover, the docking mode of **10b** and B-RAF also confirmed its interaction with the active sites of kinases. Taken together, **10b** might be a prospective compound to be developed as a promising Sorafenib-PEG-DGL.

## Figures and Tables

**Table 1:** Antiproliferation activities against seven cancer cell lines for **10a**–**c** and **15a**–**b**.

	IC_50_ (μM)						
Compound	HepG2	Huh7	MCF-7	MDA-MB-231	Hela	A549	B16F10
10a	70.67	318.8	471.2	NA^a^	NA	287.3	NA
10b	282.2	58.79	159.3	294.6	63.67	446.6	NA
10c	90.1	88.35	268.1	329.9	232.7	NA	449.3
15a	80.14	NA	NA	NA	NA	46.23	NA
15b	NA	NA	NA	NA	NA	NA	NA
**Sorafenib**	24.07	19.49	37.23	12.26	49.46	17.44	17.34

a: NA for IC_50_> 500 μM.

**Table 2:** Antiproliferation activities for **10a**–**c** and **15a**–**b** in nontumorigenesis cells.

IC_50_ (μM)	10a	10b	10c	15a	15b	Sorafenib
HUVEC	582.6	197.3	329.4	320.5	1246	35.45
293T	643.3	135.3	454.4	1576	559.4	16.07

## Supplementary Materials

Supplementary Material includes NMR spectra of target compounds **10a**–**b** and **15a**–**b**.

## References

[1] Hubbard S.R., Till J.H. (2000). Protein tyrosine kinase structure and function. Annu. Rev. Biochem..

[2] Whisler R.L., Bagenstose S.E., Newhouse Y.G., Carle K.W. (1997). Expression and catalytic activities of protein tyrosine kinases (PTKs) Fyn and Lck in peripheral blood T cells from elderly humans stimulated through the T cell receptor (TCR)/CD3 complex. Mech. Ageing Dev..

[3] Al-Obeidi F.A., Lam K.S. (2000). Development of inhibitors for protein tyrosine kinases. Oncogene.

[4] Lowinger T.B., Riedl B., Dumas J., Smith R.A. (2002). Design and discovery of small molecules targeting raf-1 kinase. Curr. Pharm. Des..

[5] Gril B., Palmieri D., Qian Y., Anwar T., Ileva L., Bernardo M., Choyke P., Liewehr D.J., Steinberg S.M., Steeg P.S. (2011). The B-Raf status of tumor cells may be a significant determinant of both antitumor and anti-angiogenic effects of pazopanib in xenograft tumor models. Plos ONE.

[6] Adnane L., Trail P.A., Taylor I., Wilhelm S.M. (2006). Sorafenib (BAY 43-9006, Nexavar), a dual-action inhibitor that targets RAF/MEK/ERK pathway in tumor cells and tyrosine kinases VEGFR/PDGFR in tumor vasculature. Methods Enzymol..

[7] Wilhelm S., Carter C., Lynch M., Lowinger T., Dumas J., Smith R.A., Schwartz B., Simantov R., Kelley S. (2006). Discovery and development of sorafenib: A multikinase inhibitor for treating cancer. Nat. Rev. Drug Discov..

[8] Wilhelm S.M., Carter C., Tang L., Wilkie D., McNabola A., Rong H., Chen C., Zhang X., Vincent P., McHugh M. (2004). BAY 43-9006 Exhibits Broad Spectrum Oral Antitumor Activity and Targets the RAF/MEK/ERK Pathway and Receptor Tyrosine Kinases Involved in Tumor Progression and Angiogenesis. Cancer Res..

[9] Liu L., Cao Y., Chen C., Zhang X., McNabola A., Wilkie D., Wilhelm S., Lynch M., Carter C. (2006). Sorafenib blocks the RAF/MEK/ERK pathway, inhibits tumor angiogenesis, and induces tumor cell apoptosis in hepatocellular carcinoma model PLC/PRF/5. Cancer Res..

[10] Wan P.T., Garnett M.J., Roe S.M., Lee S., Niculescu-Duvaz D., Good V.M., Jones C.M., Marshall C.J., Springer C.J., Barford D. (2004). Mechanism of activation of the RAF-ERK signaling pathway by oncogenic mutations of B-RAF. Cell.

[11] Qin A.F., Li Y., Song H.R., Chen X.G., Jin X.F., Wang K., Zhang L.J., Huo L.C., Feng Z.Q. (2012). Design, synthesis and antitumor activity of sorafenib analogues containing 2-picolinylhydrazide moiety. Acta Pharm. Sin..

[12] Trott O., Olson A.J. (2010). AutoDock Vina: Improving the speed and accuracy of docking with a new scoring function, efficient optimization, and multithreading. J. Comput Chem.

[13] Mutlu H., Ceper E.B., Li X., Yang J., Dong W., Ozmen M.M., Theato P. (2019). Sulfur Chemistry in Polymer and Materials Science. Macromol. Rapid Commun..

