# Supplementary material for: Introduction of Mercaptoethyl at Sorafenib Pyridine-2-Amide Motif as a Potentially Effective Chain to Further get Sorafenib-PEG-DGL
Source: Molecules. 2020 Jan 28;25(3):573. doi: 10.3390/molecules25030573 (PMC7037283; doi:10.3390/molecules25030573)
Supplement: Supplementary file 1 [file molecules-25-00573-s001.pdf]

Supplementary Information

Introduction of mercaptoethyl at sorafenib pyridine-2-amide motif as a potentially effective chain to further get Sorafenib-PEG-DGL

Ke Wang[1,2,#], Kudelaidi Kuerbana[3,#], Qi Wan[1], Zhihui Yu[1], Li Ye[3,*], Ying Chen[1,*]

1 Department of Medicinal Chemistry, School of Pharmacy, Fudan University, Shanghai, 201203, China

2 Pharmaceutical Sciences Division, School of Pharmacy, University of Wisconsin-Madison, Madison, WI, 53705-2222, USA

3 Department of Microbiological & Biochemical Pharmacy, School of Pharmacy, Fudan University, Shanghai 201203, China

[#]These authors contributed equally to this work

*Correspondence: yelil@fudan.edu.cn (L.Y.); yingchen71@fudan.edu.cn (Y.C.)

Table of contents

Images of NMR spectrum of target compounds

Figure S1. ^{1}H NMR (400 MHz, DMSO-d_6) and ^{13}C NMR (151 MHz, DMSO-d_6) spectrum of **10a**.

Figure S2. ^{1}H NMR (400 MHz, DMSO-d_6) and ^{13}C NMR (151 MHz, DMSO-d_6) spectrum of **10b**.

Figure S3. ^{1}H NMR (400 MHz, DMSO-d_6) and ^{13}C NMR (151 MHz, DMSO-d_6) spectrum of **10c**.

Figure S4. ^{1}H NMR (400 MHz, DMSO-d_6) and ^{13}C NMR (151 MHz, DMSO-d_6) spectrum of **15a**.

Figure S5. ^{1}H NMR (400 MHz, DMSO-d_6) and ^{13}C NMR (151 MHz, DMSO-d_6) spectrum of **15b**.

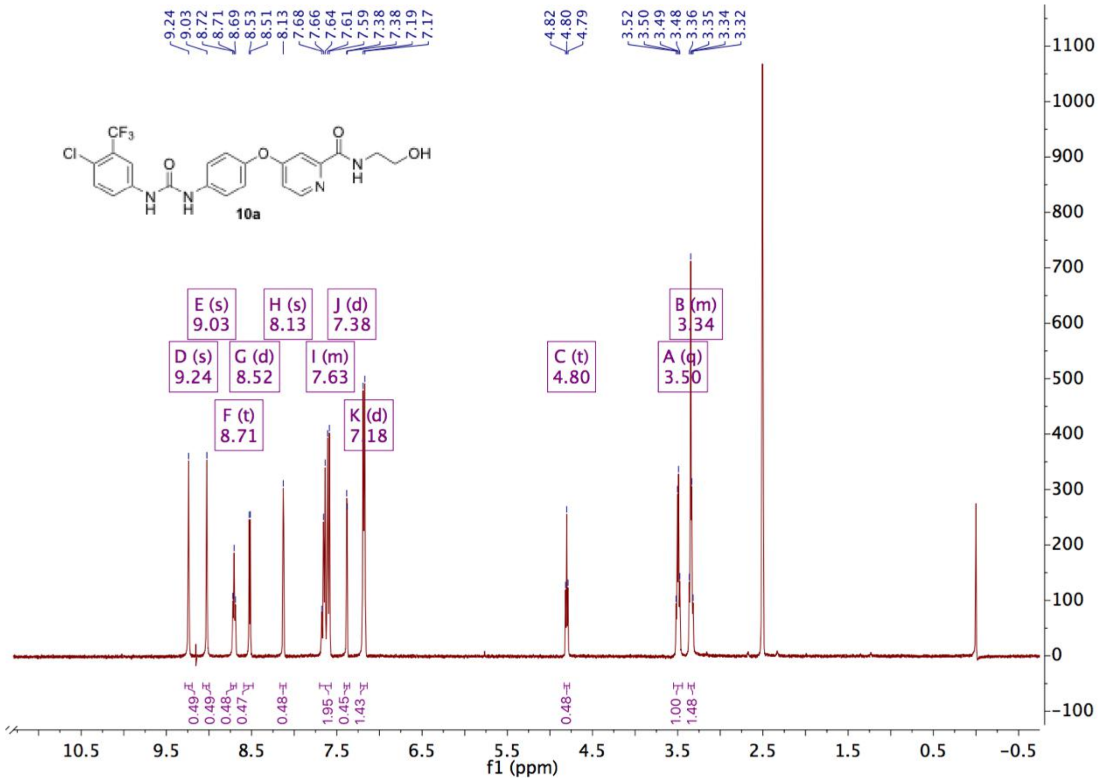

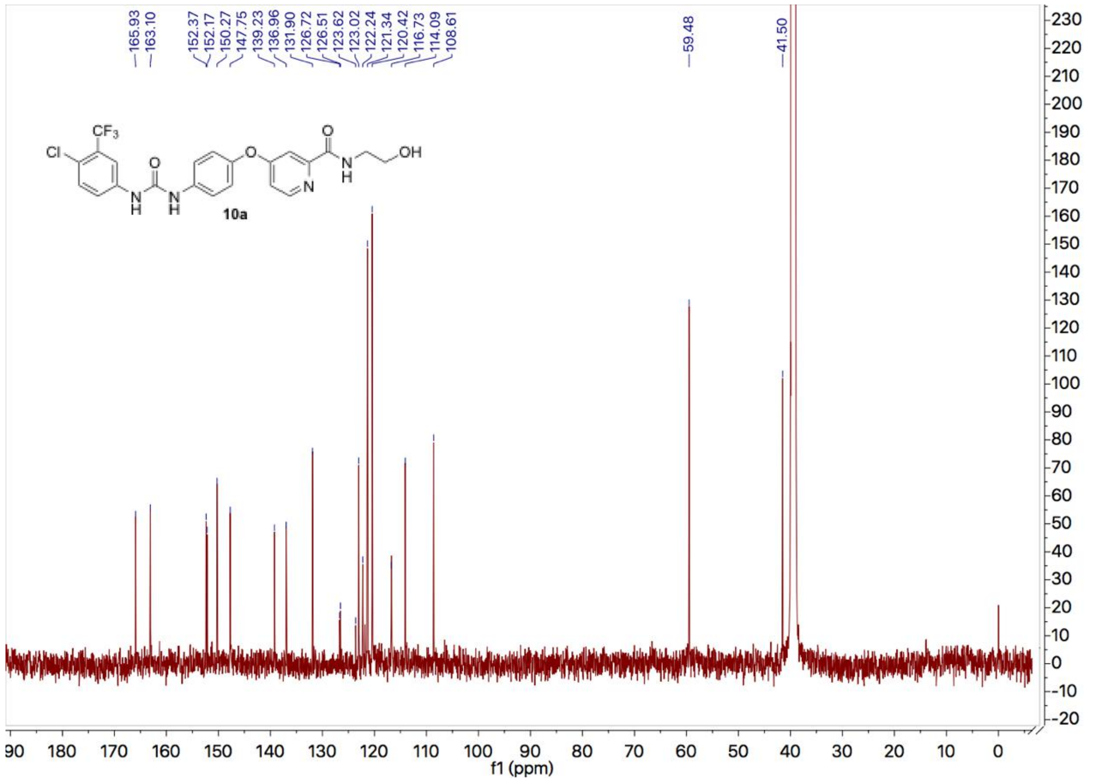

Figure S1. ^{1}H NMR (400 MHz, DMSO-d_6) and ^{13}C NMR (151 MHz, DMSO-d_6) spectrum of **10a**.

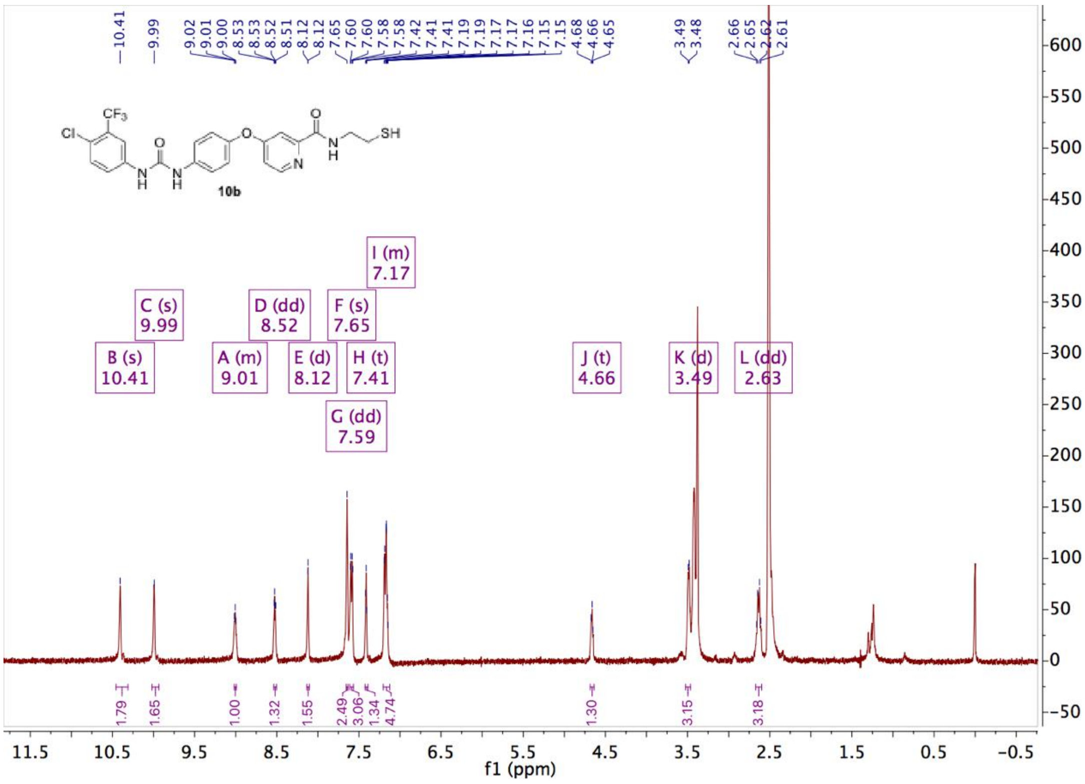

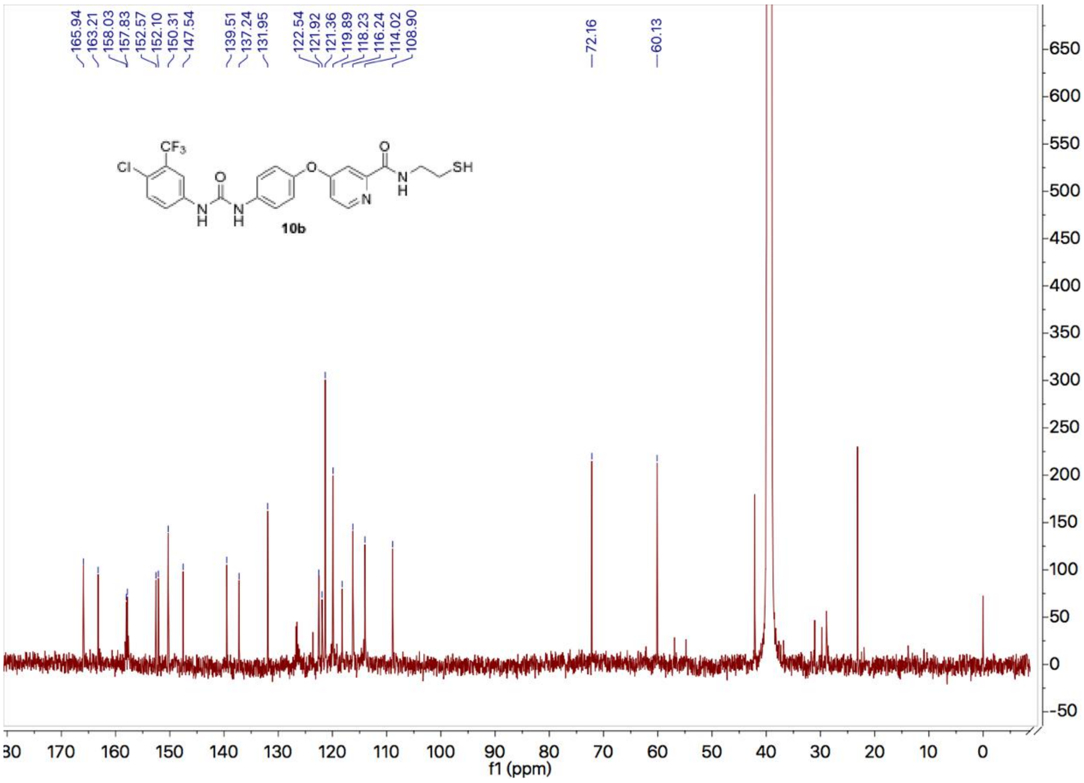

Figure S2. ^{1}H NMR (400 MHz, DMSO-d_6) and ^{13}C NMR (151 MHz, DMSO-d_6) spectrum of **10b**.

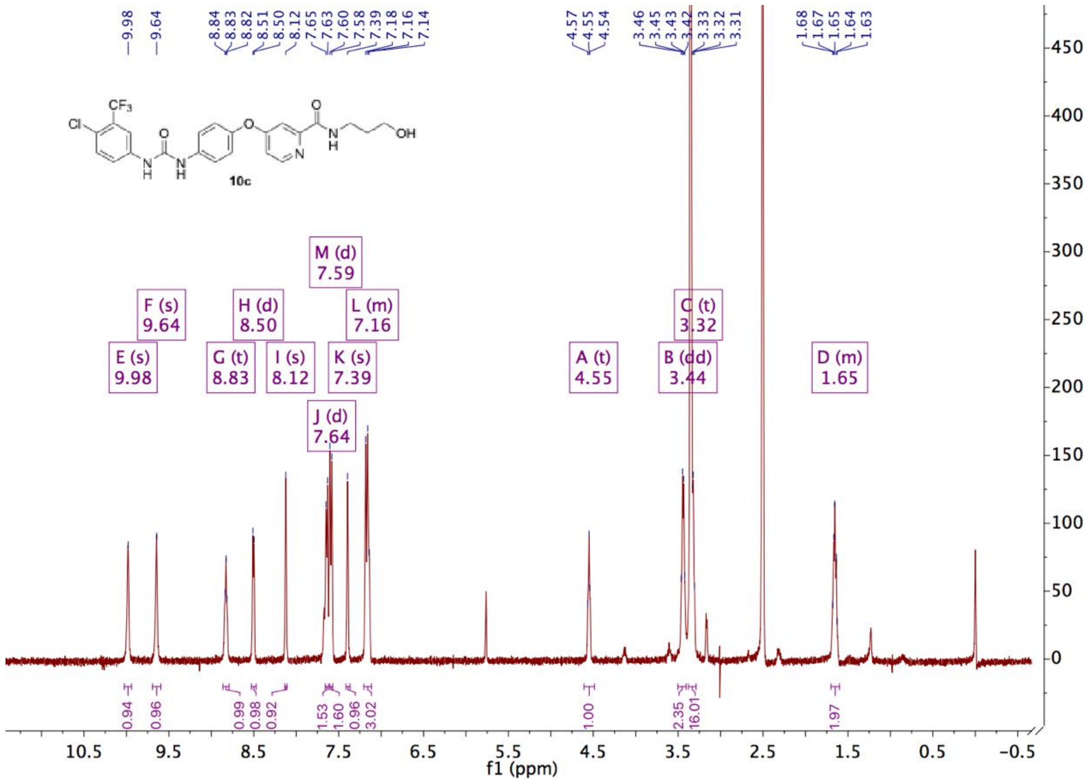

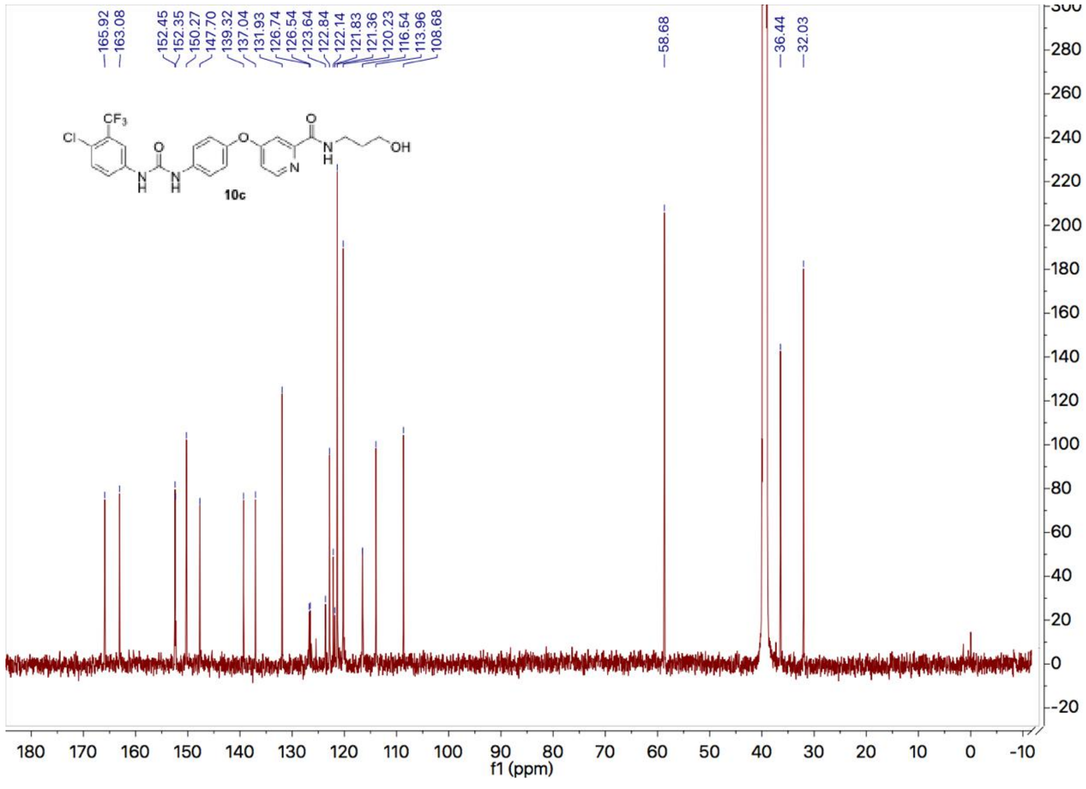

Figure S3. ^{1}H NMR (400 MHz, DMSO-d_6) and ^{13}C NMR (151 MHz, DMSO-d_6) spectrum of **10c**.

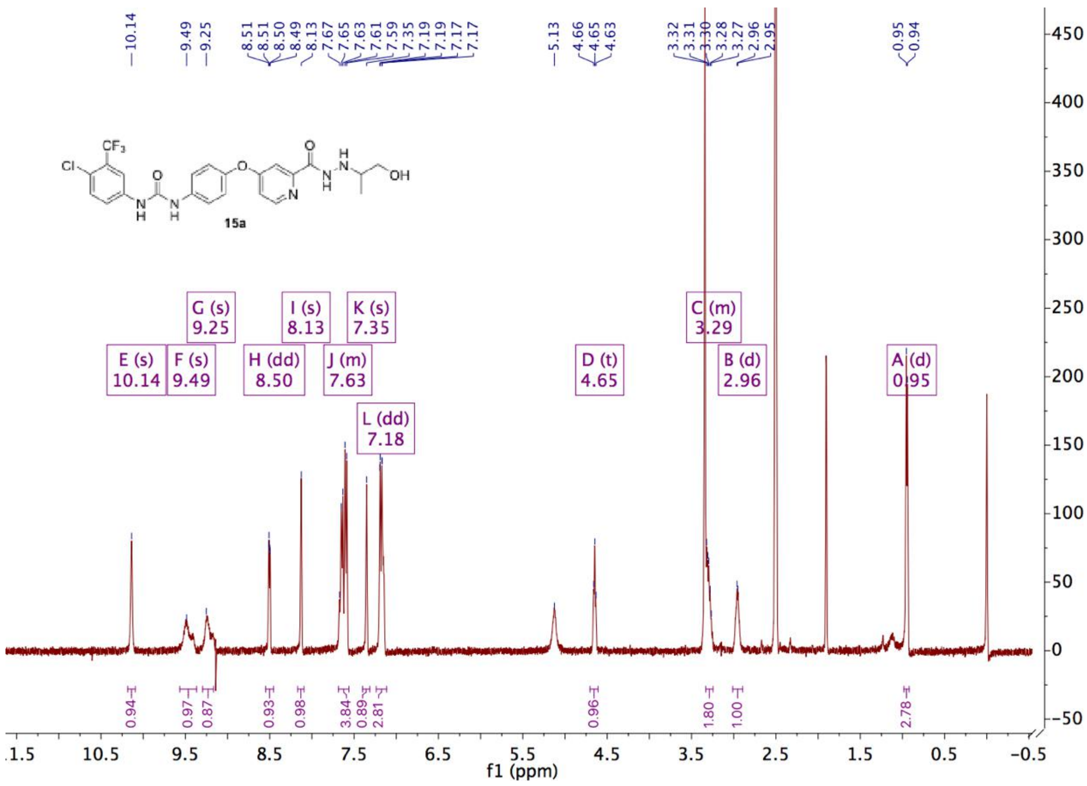

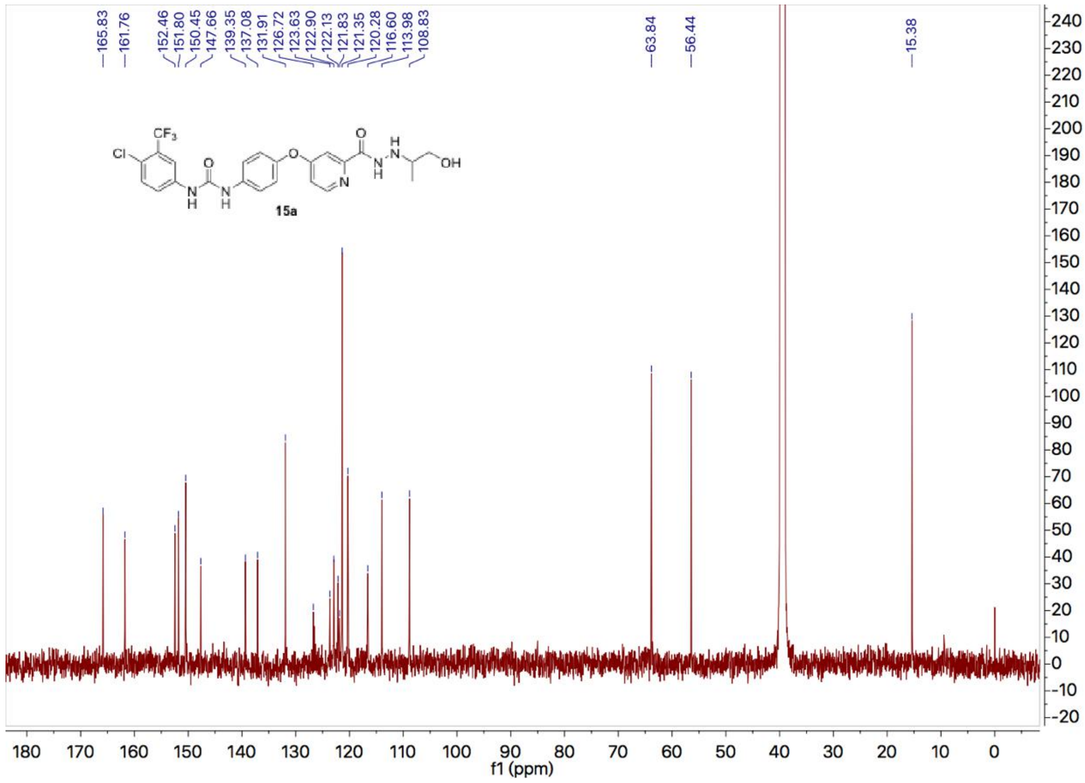

Figure S4. ^{1}H NMR (400 MHz, DMSO-d_6) and ^{13}C NMR (151 MHz, DMSO-d_6) spectrum of **15a**.

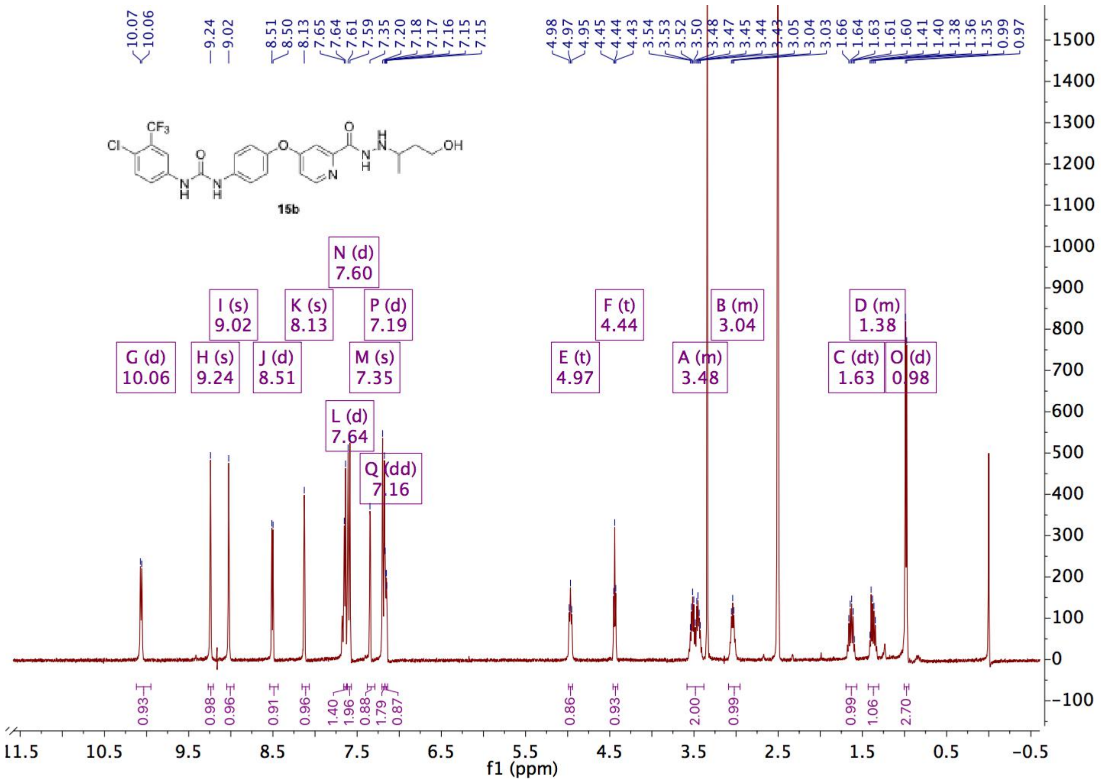

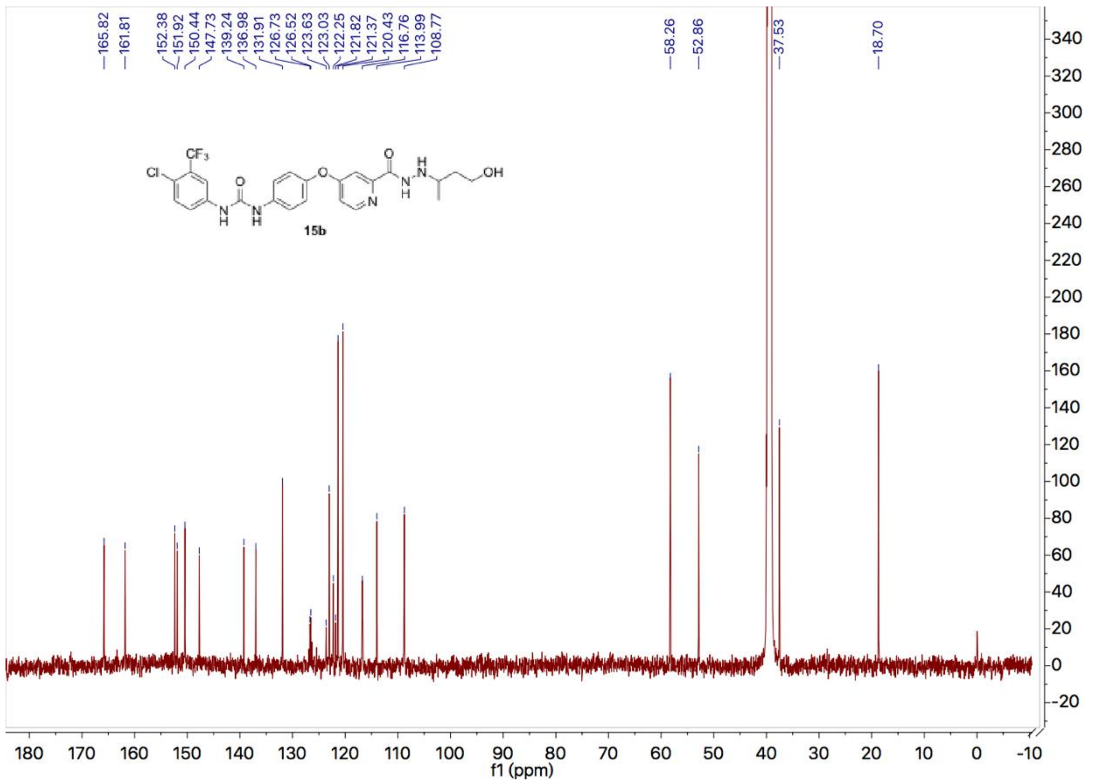

Figure S5. ^{1}H NMR (400 MHz, DMSO-d_6) and ^{13}C NMR (151 MHz, DMSO-d_6) spectrum of **15b**.
